# The Association Between Adherence to the Mediterranean Diet and Depression and Anxiety Symptoms in University Students: The Mediating Role of Lean Mass and the Muscle Strength Index

**DOI:** 10.3390/nu17020346

**Published:** 2025-01-18

**Authors:** Sofía Alfaro-González, Miriam Garrido-Miguel, Carlos Pascual-Morena, Diana P. Pozuelo-Carrascosa, Rubén Fernández-Rodríguez, José Alberto Martínez-Hortelano, Arthur E. Mesas, Vicente Martínez-Vizcaíno

**Affiliations:** 1 Health and Social Research Center, Universidad de Castilla La-Mancha, 16071 Cuenca, Spain; sofia.alfaro@alu.uclm.es (S.A.-G.); carlos.pascual@uclm.es (C.P.-M.); dianap.pozuelo@uclm.es (D.P.P.-C.); ruben.fernandez@uclm.es (R.F.-R.); arthur.emesas@uclm.es (A.E.M.); vicente.martinez@uclm.es (V.M.-V.); 2 Facultad de Enfermería, Universidad de Castilla-La Mancha, 02006 Albacete, Spain; 3 Department of Physical Education and Sports, Faculty of Sports Science, Sport and Health University Research Institute (iMUDS), University of Granada, 18016 Granada, Spain; 4 Cuidado Comunitario y Determinantes Sociales de la Salud, Enfermería, Departamento de Enfermería y Fisioterapia, Facultad de Enfermería y Fisioterapia, Universidad de Alcalá, 28801 Madrid, Spain; josealberto.martinez@uah.es; 5 Facultad de Ciencias de la Salud, Universidad Autónoma de Chile, Talca 3460000, Chile

**Keywords:** Mediterranean dietary pattern, physical fitness, mental health, young adults

## Abstract

Background/objectives: recent studies have suggested that components typical of the Mediterranean diet (MedDiet) are associated with depression and anxiety prevention. In this sense, the main objective of this study was to analyse the associations between adherence to the MedDiet and depression and anxiety symptoms and to examine whether this relationship is mediated by lean mass and the muscle strength index (MSI). Methods: a cross-sectional study (based on data obtained from the Nuts4Brain-Z study) was conducted from 2023–2024, involving 428 university students, aged 18–30 years, from a Spanish public university. Depression was assessed using the Beck Depression Inventory (BDI-II), and anxiety was assessed via the General Anxiety Disorder-7 (GAD-7) tool. Adherence to the MedDiet was assessed using the MEDAS questionnaire. Lean mass was assessed via bioimpedance, and MSI was measured via a dynamometer. ANCOVA models were used to test the mean differences in depression and anxiety scores using MEDAS categories (low adherence < 9 points vs high adherence ≥ 9 points). Serial multiple mediation models, adjusted for the main confounders, were used to explore the role of lean mass and MSI in the relationships between adherence to the MedDiet and depression and anxiety symptoms. Results: university students with high adherence to the MedDiet exhibited lower scores for depression and anxiety symptoms (*p* < 0.05) than did students with low adherence to the MedDiet. The mediation analysis preliminarily revealed that both lean mass and MSI acted as mediators of the relationships between adherence to the MedDiet and depression and anxiety. Conclusion:adherence to the MedDiet in university students per se does not appear to have a direct effect on depression and anxiety symptoms because these associations are partially (for depression) or entirely (for anxiety) explained by lean mass and MSI.

## 1. Introduction

According to the World Health Organization (WHO), mental health is defined as “a state of mental well-being that enables people to cope with the stresses of life, realize their abilities, learn well and work well, and contribute to their community” [1]. According to the Global Burden Diseases, Injuries, and Risk Factors study between 1990 and 2019, depression and anxiety are the most disabling mental disorders worldwide [2], and after the COVID-19 pandemic, cases of depression (27.6%) and anxiety (25.6%) increased [3], with women and younger people being especially affected. However, depression and anxiety are multifactorial, of which 40% are caused by genetic influences [4]. Other causes could include individual factors (personality, chronic pathologies), including familiar or social environments, especially in the university period, that could be a difficult time for young people, as many students leave their family homes to live in university residences or shared apartments, especially during their first year of education [5]. Other modifiable factors could include social stressors (social media, social insolation), as well as lifestyle choices, such as unhealthy diet, physical fitness, sleep quality, the academic environment, or alcohol abuse, among others, which can influence the mental health of young people, leading to poor mental health, especially the development of anxiety or depression [4,6,7].

In Spain, next to Mediterranean areas such as Greece, Italy, and Turkey, the traditional diet of the population is the Mediterranean diet (MedDiet). The latest MedDiet recommendations are linked to sustainability. In this regard, the MedDiet pyramid mentioned, compared with the pyramid described in 2010 [8], is characterized by an intake of fruits, cereals with whole grains, legumes, and olive oil at every meal; a daily consumption of olives/nuts/seeds, spices, and dairy; a weekly consumption of white meat, fish/seafood, and eggs; and occasional intake of red meat, processed meat, and sweets [9]. The MedDiet is a plant-forward diet rich in monounsaturated fatty lipids, plant-based proteins, vitamins, and antioxidant compounds such as polyphenols, minerals, fibres, and a balanced n-6:n-3 fatty acid ratio [8,10]. This diet has also been highly explored [11] because it is related to a lower risk of noncommunicable diseases such as cardiovascular diseases [12], cancer [13], type 2 diabetes mellitus [14], and mental health diseases [15].

In addition to pharmacotherapy and psychotherapy, nutritional psychiatry, which is considered a promising approach for managing mental health through diet and nutrition, is needed to improve mental health [16]. Accordingly, the MedDiet has been associated with a reduction in depression symptoms [17,18,19,20]. Several ways in which diet and its components may influence depression and anxiety have been identified [21]. In this sense, recent studies have suggested that an increased intake of polyphenols, which are present in vegetables, fruits, or nuts, among other components typical of the MedDiet, is associated with depression and anxiety prevention [15,22]. In this context, consuming a healthy diet is related to a better lifestyle and better mental health. In addition, recent studies have also demonstrated a bidirectional relationship between excessive levels of anxiety or depression leading to the use of dysfunctional emotional regulation strategies, such as disordered eating behaviours (e.g., binge eating), leading to further eating disorder psychopathology [23].

According to a meta-analysis [24,25], strength exercise can reduce depressive symptoms, regardless of the participants’ health or exercise status. The mechanisms that could be involved in this relationship include myokines and cytokines, which are liberated by muscle contractions [26], and are also related to physical fitness levels and habits, which improve depression and anxiety symptoms. However, further studies are needed to verify this relationship in young adults, as most of them are focused on the older population [24,25,27,28].

Adherence to the MedDiet has been consistently related to other beneficial lifestyle indicators, such as body composition and muscle strength [11,29]. Thus, it is reasonable to assume that such benefits in mental health are due, at least in part, to a clustering effect of healthy behaviours [7,30,31]. For this reason, it is essential to clarify whether the improvement in mental health is associated with better body composition and muscle strength or with adherence to the MedDiet itself. Therefore, this study aimed to analyse the relationships between adherence to the MedDiet and depression and anxiety symptoms and to determine whether this relationship is mediated by lean mass and the muscle strength index (MSI) in a sample of 18- to 30-year-old university students.

## 2. Materials and Methods

### 2.1. Study Design and Population

This is a cross-sectional study based on data obtained from the Nuts4Brain-Z (N4B-Z) study, which involves a sample of university students from Universidad de Castilla-La Mancha (UCLM) in Cuenca, Spain.

The N4B-Z project was designed to research the association between the consumption of nuts and depression. We determined a minimum sample size of 454 university students via GRANMO 7.12 statistical software to detect a mean difference of 0.3 ± 2.04 units on the Center for Epidemiological Studies Depression Scale (CESD-R), with a statistical power of 0.8 and an alpha risk of 0.05. Data collection was completed during the months of September and December of the 2023–2024 academic year. The initial sample included 463 young adults (100.0%). However, in the present study, we analysed data from a subsample of 428 university students (92.44%), in which all dataset variables were evaluated.

The inclusion criteria for our sample were as follows: (i) students from UCLM aged between 18 and 30 years. The study included students from the health sciences (nursing), engineering, and social and legal sciences departments.

### 2.2. Ethics Approval and Consent to Participate

The N4B-Z protocol was approved by the Research Ethics Committee of the *Hospital Virgen de la Luz*, Cuenca (Spain) (report number 2023/PI1323), and conformed with the Declaration of Helsinki. The conditions and characteristics of the study were disclosed, and signed consent to participate in the present study was obtained from all study participants.

### 2.3. Study Variables

#### 2.3.1. Depression (Dependent Variable)

Depressive conditions in university students were assessed with the Beck Depression Inventory (BDI-II) [32]. This self-reporting questionnaire, which is widely used, measures the severity of depression using 21 self-rating scales with multiple choices about how the subject has been feeling in the last two weeks in regards to depression. Each item is measured on a 4-point Likert scale, with 0 = “normal” to 3 = “most severe”. The maximum total score for all 21 items is 63. Scores of 0–13 indicate minimal depression, 14–19 indicate mild depression, 20–28 indicate moderate depression, and 29–63 indicate severe depression.

#### 2.3.2. Anxiety (Dependent Variable)

The General Anxiety Disorder-7 (GAD-7) tool was used to assess the severity of anxiety disorders in the sample [33], as registered in the Diagnostic and Statistical Manual of Mental Disorders (Fifth Edition) (DSM-5) [34]. This questionnaire is also a self-administered questionnaire in which the scores range from 0–21, with responses measured using a 4-point Likert scale. The final score is categorized into four groups: minimal (04), mild (5 to 9), moderate (10 to 14), and serious anxiety (15 to 21).

#### 2.3.3. Adherence to the Mediterranean Diet (Independent Variable)

We used the Mediterranean diet adherence screener (MEDAS) [35] questionnaire to evaluate adherence to the MedDiet designed in the Prevención con Dieta Mediterránea (PREDIMED) study. The MEDAS is a commonly used Spanish-validated scale with 14 items, each ranging from 0 to 1, with a total score of 14 points. It is categorized into two different categories, i.e., scores < 9 indicate low adherence to the MedDiet, and scores from 9 to 14 indicate good adherence to the MedDiet. These items refer to the frequency of daily and weekly food consumption of olive oil, fruits (including juice), vegetables, animal fats, red meat, carbonated beverages, red wine, legumes, fish/seafood, nuts, sweets and pastries, traditional dishes, cooking-fat, and meat.

#### 2.3.4. Potential Mediators: Lean Mass and Muscle Strength Index

The lean mass and fat-free mass were obtained twice under controlled temperature and humidity conditions, with participants shoeless and fasting, using a bioimpedance analysis system (Tanita MC-780 MA). Both variables were measured in kilograms (kg).

The MSI was determined as the sum of the standardized *z* score of handgrip/ weight and the *z* score of the standing long jump. Handgrip strength was evaluated via a dynamometer (TKK 5401 Grip-D; Takey, Tokyo, Japan). The participants squeezed the dynamometer gradually and continuously as hard as possible for at least two seconds. The average of four measurements (two times each hand) was reported in kilograms. The standing long jump was measured in centimetres. The students stood behind the line jump line and jumped up three times, with both feet in a stable position. The best distance of the three attempts was recorded and used in the analysis.

#### 2.3.5. Covariates

Self-reported information was obtained for gender (female and male) and birth date. Age was assessed from birth date. Height was calculated twice via a stadiometer (SECA Model 213; Vogel & Halke; Hamburg, Germany; precision, 0.1 cm; range, 20–205 cm). An electronic scale (SECA Model 869; Vogel & Halke; Hamburg, Germany; precision, 100 g; range, 2–250 kg) was used to assess weight as the average of two measurements in kilograms. Additionally, body mass index (BMI) was calculated as weight divided by the square of height (kg/m^2^). BMI was categorized according to the WHO criteria into three groups: normal weight (18.5 ≤ BMI ≤ 24.9), overweight (25 ≤ BMI ≤ 29.9), and obese (BMI ≥ 30) [36].

The level of parental education was self-reported for both parents [37]. We included in the analyses the highest level of education for both parents.

Alcohol and tobacco risk was assessed via the alcohol, smoking, and substance involvement screening test (ASSIST) [38]. This questionnaire calculates the risk score for tobacco into three levels—low risk (0–3 points), moderate risk (4–26 points), and high risk (>27 points); and for alcohol into three levels—low risk (0–10), moderate risk (11–26), and high risk (>27 points). Total energy intake (Kcal) was determined via the Food Frequency Questionnaire (FFQ-137 items) [39].

These variables were selected as potential confounding variables based on previous evidence of their associations with both the Mediterranean diet and mental health and as previously used in similar articles [40,41,42,43,44,45].

### 2.4. Statistical Analysis

Either the Student’s *t* test (continuous variables) or the Chi-squared test (categorical variables) was used to analyse the descriptive characteristics of the study sample by MEDAS categories. The normal distribution of continuous variables was examined via both statistical (Kolmogorov–Smirnov test) and graphical methods (normal probability plots). The participants did not show significant differences according to their fields of study (health sciences or non-health sciences) regarding the main variables analysed in the study. Therefore, we decided to analyse the sample as a whole so as not to lose statistical power (Table S1).

Partial correlation coefficients were calculated to examine the relationships among the MEDAS score, fat mass, lean mass, MSI, handgrip strength, standing long jump, and depression and anxiety symptoms. Subsequently, analysis of covariance (ANCOVA) models were used to test the mean differences in depression and anxiety as dependent variables by MEDAS categories (low vs good adherence to the MedDiet), controlling for gender, age, total energy, alcohol consumption, tobacco consumption, level of parental education, and potential mediators (lean mass and MSI) in Model 1.

Finally, we performed serial multiple mediation analyses via the PROCESS SPSS macro, version 4.2 [46], to determine whether lean mass and MSI act as mediators in the relationships between adherence to the MedDiet (MEDAS score) and depression and anxiety symptoms. For these analyses, we selected a serial multiple mediation model using 5000 bootstrap [47] samples to calculate the confidence intervals (95% CIs), with lean mass as the first mediator and MSI as the second mediator. The mediation model used (Model 6 with two mediators) explores the total (c) and direct effects (a1, a2, b1, b2, d, and c′) that indicate the unstandardized regression coefficient and significance between adherence to the MedDiet and depression and anxiety symptoms. Additionally, this model examines three indirect effects (IE1, IE2, and IE3) that indicate the change in depression and anxiety for each unit change regarding adherence to the MedDiet, which is mediated by lean mass and the MSI. The IEs were considered significant when the 95% CI did not contain zero [48]. The directions of the direct and indirect effects in each mediation model are highlighted with colours.

The mediation analysis was adjusted for gender, age, total energy, alcohol consumption, tobacco consumption, and level of parental education.

All analyses were performed via IBM SPSS Statistics software, version 28.0 (IBM Corp., Armonk, NY, USA), and *p* < 0.05 was considered to indicate significance.

## 3. Results

The characteristics of the university students are described in Table 1. A total of 428 participants (71% female) with a mean age of 21.1 ± 3.0 years, were analysed. Only 26.4% of the samples displayed good adherence to the MedDiet. The mean MEDAS score was 7.3 ± 1.9. University students with good adherence to the MedDiet had lower values of alcohol and tobacco consumption, higher values of total lean mass and muscle strength indicators, and lower values of depression and anxiety symptoms than did those with low adherence to the MedDiet.

Table 2 shows the partial correlations of the study variables. Adherence to the MedDiet (MEDAS score) was negatively associated with depression (r = −0.223; *p* < 0.001) and anxiety (r = −0.118; *p* < 0.05) and positively associated with lean mass (r = 0.113; *p* < 0.05), MSI (r= 0.118; *p* < 0.05), handgrip strength (r = 0.097; *p* < 0.05), and standing long jump (r = 0.119; *p* < 0.05). Lean mass was negatively associated with depression (r = −0.108; *p* < 0.05) and anxiety (r = −0.209; *p* < 0.001) and positively associated with MSI (r = 0.737; *p* < 0.001), handgrip strength (r = 0.824; *p* < 0.001), and standing long jump (r = 0.527; *p* < 0.001). MSI was also negatively associated with depression (r = −0.246; *p* < 0.001) and anxiety symptoms (r = −0.253; *p* < 0.001).

**Figure 1:**
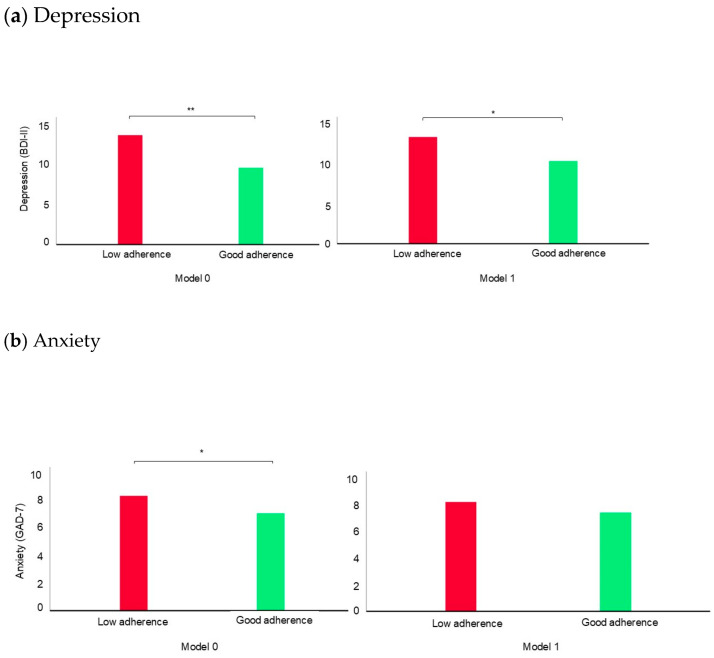
The mean differences between (**a**) depression (BDI-II) and (**b**) anxiety (GAD-7) according to adherence to the MedDiet (MEDAS) categories (scores from 9 to 14 indicate good adherence to the MedDiet). Model 0: crude data; Model 1: model adjusted by gender, age, total energy, alcohol consumption, tobacco consumption, level of parental education, lean mass, and MSI. * *p* < 0.05; ** *p* < 0.001. BDI-II: Beck Depression Inventory, second edition; GAD: generalized anxiety disorder; EI: energy intake; MEDAS: Mediterranean diet adherence screening tool; MSI: muscle strength index.

Figure 1 shows the mean differences between depression and anxiety symptoms according to the MedDiet (MEDAS) categories. The results of Model 0, shown in Figure 1a,b, indicate that university students who show good adherence (vs low adherence) to the MedDiet have significantly lower levels of depression and anxiety symptoms. However, when we adjusted for potential confounders, including lean mass and MSI (Model 1), part (Figure 1a) or all (Figure 1b) of the statistical significance reduces and disappears, respectively, suggesting a potential mediating effect of lean mass and MSI on this relationship.

### Mediation Analysis

**Figure 2:**
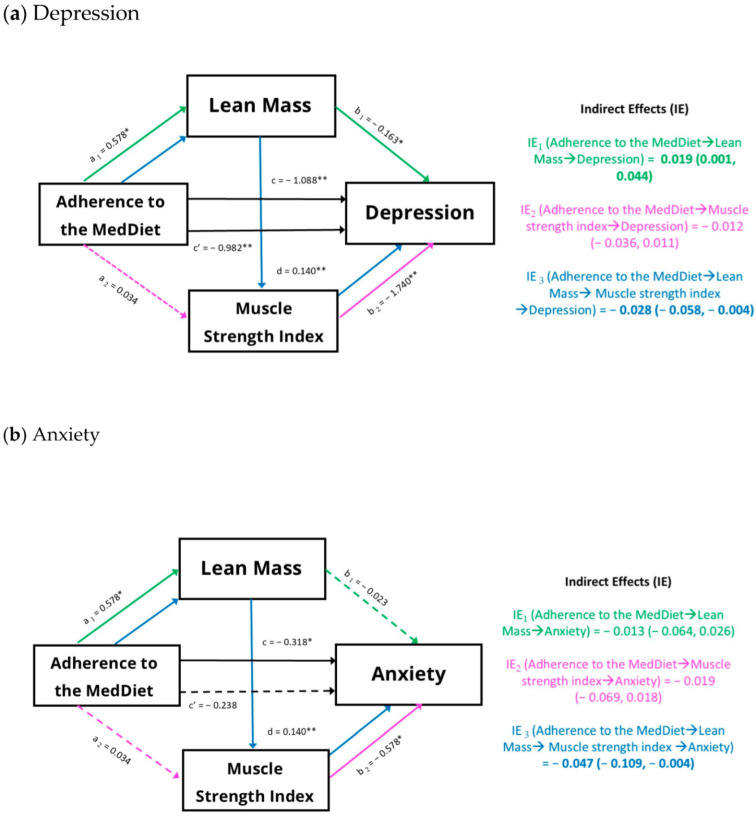
Mediation analysis of the associations between adherence to the MedDiet (MEDAS score) and (**a**) depression (BDI-II) and (**b**) anxiety (GAD-7) symptoms. Serial multiple mediation models were used, with lean mass and the muscle strength index as mediators, controlling for gender, age, total energy, alcohol consumption, tobacco consumption, and level of parental education. The values for the a1, a2, b1, b2, d, c, and c’ paths are shown as unstandardized regression coefficients (standard errors). IE1, IE2, and IE3 are represented as unstandardized regression coefficients (95% confidence intervals). Continuous lines (pathways) and bold values (IEs) designate a statistically significant effect. * *p* < 0.05; ** *p* < 0.001. BDI-II: Beck Depression Inventory, second edition; GAD: generalized anxiety disorder; IE: indirect effect; MEDAS: Mediterranean diet adherence screening tool.

We assessed the mediating role of lean mass and MSI in the relationships between adherence to the MedDiet and depression and anxiety symptoms (Figure 2). The total effect indicated that greater adherence to the MedDiet was associated with fewer symptoms of depression and anxiety (path c). However, in the last equation, this association was reduced (for depression) or disappeared completely (for anxiety) when the mediators (lean mass and MSI) were incorporated into the model (path c’). In this sense, lean mass and MSI partially (Figure 2a) or entirely (Figure 2b) mediated, respectively, the associations between adherence to the MedDiet and depression and anxiety symptoms. Indirect effect 3 (IE_3_) (Figure 2) demonstrated that greater adherence to the MedDiet was associated with increased lean mass, which in turn was linked to improvements in MSI and consequently, a reduction in symptoms of depression and anxiety.

## 4. Discussion

This cross-sectional study analysed the associations of adherence to the MedDiet with depression and anxiety symptoms in Spanish university students and investigated the mediating roles of lean mass and MSI in this relationship. Our study supports the idea that (i) university students with greater adherence to the MedDiet displayed fewer symptoms of depression and anxiety and that (ii) lean mass and MSI mediated the associations between adherence to the MedDiet and depression and anxiety symptoms.

### 4.1. Adherence to the MedDiet and Depression and Anxiety Symptoms

Various studies have shown the importance of healthy diets, such as the MedDiet, in the prevention and control of depression or anxiety [49]. Our findings preliminarily showed that university students who have higher adherence to the MedDiet exhibit lower depression and anxiety levels. These results are in line with those of the systematic reviews and meta-analyses of observational studies [19,50,51,52], which have shown that adherence to the MedDiet is inversely associated with depression and anxiety symptoms. Furthermore, different randomized controlled trials (RCTs) [53,54,55,56] have reported this positive relationship, but further RCTs are needed to confirm this association.

This relationship could be explained by elements of the MedDiet pattern. Nuts, fish, olive oil, vegetables, and fruits are the most influential elements of brain health status [50], as presented in the MedDiet. These foods are rich in vitamins such as vitamins C, B12, and folate, which are essential for the production of neurotransmitters such as serotonin and dopamine [57,58]; minerals such as magnesium, zinc, and iron [59]; polyunsaturated fatty acids (PUFAs), such as omega-3 [60]; and polyphenols [15], which are related to better health mental status. Polyphenols [15], next to the gut microbiota [61], have been widely studied because they are positively associated with depression and anxiety. A recent systematic review [15] revealed that different types of polyphenols (such as soy isoflavones, tea and cocoa flavanols, curcumin, coffee hydroxycinnamic acid, walnut flavanols, citrus flavanones, and the stilbene resveratrol) are the most effective against depression. However, polyphenols have poor intestinal absorption but are metabolized by the gut microbiota, especially in the digestive tract. In addition to these relationships, further RCTs are needed to determine the cause–effect relationship between polyphenols and depression. The gut microbiota, as mentioned above, plays an important role in mental health [61,62,63]. The key to gut microbiota eubiosis and better mental health is a healthy diet, such as the MedDiet [61]. In contrast, dysbiosis is associated with depression and anxiety, but further studies are needed to verify this relationship. Gut microbiota eubiosis is related to neurotransmitter production, reduced gastrointestinal inflammation, and strengthening of the gut barrier [61]. Conversely, dietary patterns characterized by a high intake of ultra-processed foods rich in saturated fats, free sugars, additives, and salt are associated with increased depression and anxiety risk [64,65,66].

Depression and anxiety are leading disorders in the global burden of disease. Specifically, our sample of young adults presented high percentages of depression and anxiety symptoms (37.1% and 70.8%, respectively). These results are greater than those from a recent meta-analysis, which revealed that the prevalence of depression and anxiety symptoms among university students worldwide was 33.6% and 39.0%, respectively [67].

Otherwise, only 26.4% of our university students showed good adherence to the MedDiet. In this context, university students are vulnerable, as many of them consume unhealthy rather than healthy diets, such as the MedDiet [49], which suggests a greater risk of depression and anxiety symptoms.

### 4.2. Adherence to the MedDiet, Lean Mass and MSI

A recent systematic review and meta-analysis revealed that high adherence to the MedDiet (compared with low adherence) was associated with higher physical fitness levels in adults [29], although the relationship between the MedDiet and MSI remains inconclusive; thus, further studies are needed to compare adherence to the MedDiet and MSI. Our findings in university students are in line with the results of this review, which demonstrated that greater adherence to the MedDiet was associated with increased lean mass, which in turn was linked to improvements in MSI.

The MedDiet contains the main nutrients needed to achieve ideal body composition and physical fitness. It is rich in unsaturated fatty acids, essential amino acids, and antioxidants from animal- and plant-based diets. However, proteins are important macronutrients that increase lean mass and muscle strength [68]. The MedDiet is characterized by being a source of high-quality, mostly plant-based proteins. We can identify 20 dietary amino acids, but only 9 (phenylalanine, valine, tryptophan, threonine, isoleucine, methionine, histidine, leucine, and lysine) are considered essential and must be obtained through the human diet because they cannot be synthesized. Animal-based proteins contain essential amino acids, but some essential amino acids are not found in plant-based proteins [69,70]. Compared with plant-based proteins, animal-based proteins (meat, eggs, and dairy) are generally better for muscle synthesis. This is primarily due to its relatively high content of essential amino acids, especially leucine, which is crucial for stimulating muscle protein synthesis and lean mass [71]. However, plant-based protein has greater fibre content, which improves the gut microbiota; it is anti-inflammatory because of the presence of nitrates and is the best choice for the environment when compared to animal proteins [68]. Despite these findings, some studies also indicate that total protein intake is a significant factor in preserving muscle mass, and that incorporating a variety of plant-based proteins can still support muscle synthesis when it is managed correctly [68,72,73,74]. Therefore, the MedDiet can provide this high-quality protein, along with other nutrients, for MSI and lean mass synthesis and preservation.

It is important to highlight the importance of body image in young populations. Social media and society may influence young adult lifestyles and self-image. In this sense, young people are more concerned about their image, are more active, and tend to take care of both their diet and their body composition [75]. In this context, it is reasonable to assume that the beneficial effects of diet are at least partly due to a clustering effect of other healthy behaviours, such as good body composition or a good state of physical fitness.

### 4.3. Lean Mass and MSI as Mediators of Adherence to the MedDiet and Depression and Anxiety Symptoms

High adherence to the MedDiet is commonly associated with other positive healthy habits, including body composition and physical fitness indicators. However, no research to date has examined whether lean mass and MSI could act as confounding variables or as mediators of the relationships between adherence to the MedDiet and depression and anxiety symptoms. Mediation analysis, controlling for potential confounding factors, revealed that in the relationships between adherence to the MedDiet and depression and anxiety symptoms, lean mass and MSI act as mediators. In this sense, greater adherence to the MedDiet was associated with increased lean mass, which in turn was linked to improvements in MSI and consequently, to a reduction in symptoms of depression and anxiety. This reduction may be due to several mechanisms, such as the release of myokines and cytokines and the modulation of neurotransmitters such as serotonin and dopamine, which positively impact mood disorders [25]. In this context, strength exercises can provide social support and interaction, reducing feelings of isolation, a factor linked to depression and anxiety [24]. Additionally, it is related to self-confidence and self-image, as mentioned above, resulting in a better mood. These results support the idea that good body composition and muscle strength are essential factors in the association between diet and mental health.

### 4.4. Limitations

However, our study has several limitations that should be noted. First, the design of this study, a cross-sectional study, does not allow for cause–effect relationships, and the results must be interpreted with caution. To determine whether adherence to the MedDiet exerts prospective beneficial effects on depression and anxiety, longitudinal studies are needed. Second, these results are based on university students and therefore, cannot be extrapolated to the overall population. Third, dietary variables, depression, and anxiety were assembled through a self-completed questionnaire, which might have resulted in some degree of measurement error because of recall and information biases. Fourth, this study was restricted to one city in a province of Spain; thus, caution should be used.

## 5. Conclusions

Our analyses suggest that greater adherence to the MedDiet is related to fewer depression and anxiety symptoms, indicating that lean mass and muscle strength play key roles in mediating the cross-sectional associations between diet quality and depression and anxiety symptoms. Public health must reinforce strategies for preventing depression and anxiety in young adults and strengthen the promotion of a MedDiet and physical activity, including not only aerobic exercise but also muscle strength exercises, for the prevention of mental health disorders, such as through the employment of public health campaigns, mental health interventions, or free fitness spaces and programs. However, prospective observational and experimental studies are needed to study the importance of the MedDiet for muscle strength, and lean mass is related to depression and anxiety in this population.

## Figures and Tables

**Table 1:** Descriptive characteristics of the study sample according to adherence to the MedDiet.

	Total (n = 428)	Adherence to the MedDiet		*p* Value
		Low Adherence (n = 315)	Good Adherence (n = 113)	
Age (years)	21.1 ± 3.0	20.9 ± 2.9	21.2 ± 3.5	0.229
Female (%)	304 (71.0)	226 (71.7)	78 (69.0)	
Parental education—university degree (%)	147 (34.3)	107 (34.0)	40 (35.4)	0.505
Risk of alcohol use (%)				
Low risk	350 (81.8)	251 (79.7)	99 (87.6)	0.159
Moderate/High risk	78 (18.2)	64 (20.3)	14 (12.4)	
Alcohol total score	6.1 ± 5.6	6.4 ± 6.0	5.2 ± 4.6	**0.006**
Risk of tobacco use (%)				
Low risk	267 (62.4)	184 (58.4)	83 (73.5)	**0.009**
Moderate/High risk	161 (37.6)	131 (41.5)	30 (26.5)	
Tobacco total score	5.3 ± 7.4	6.0 ± 7.9	3.5 ± 5.6	**<0.001**
BMI (kg/m^2^)	23.2 ± 4.4	23.3 ± 4.5	23.1 ± 4.2	0.650
Weight status				
Underweight (%)	41 (9.6)	30 (9.5)	11 (9.7)	0.974
Normal weight (%)	271 (63.3)	198 (62.9)	73 (64.6)	
Overweight/obesity (%)	116 (27.1)	87 (7.6)	29 (25.7)	
Fat mass (kg)	17.4 ± 8.3	17.6 ± 8.4	17.0 ± 8.2	0.264
Total lean mass (kg)	47.6 ± 10.0	44.7 ± 9.5	46.5 ± 9.6	**0.046**
Muscle Strength Index (MSI) ^a^	0.0 ± 1.8	−0.05 ± 1.8	0.3 ± 1.9	**0.031**
Handgrip strength (kg)	26.3 ± 8.6	25.8 ± 8.5	27.5 ± 8.8	**0.040**
Standing long jump (cm)	137.1 ± 33.9	135.3 ± 32.8	142.2 ± 36.3	**0.032**
Energy intake (kcal)	2831.4 ± 1991.8	2771.4 ± 1948.2	2998.9 ± 2108.3	0.149
MEDAS score (0–14)	7.3 ± 1.9	6.5 ± 1.3	9.7 ± 0.9	**<0.001**
Beck Depression Inventory (BDI-II) (%)				
No depression	269 (62.9)	182 (57.8)	87 (77.0)	**0.003**
Mild depression	77 (18.0)	66 (21.0)	11 (9.7)	
Moderate depression	51 (11.9)	40 (12.7)	11 (9.7)	
Severe depression	31 (7.2)	27 (8.6)	4 (3.5)	
BDI-II total score	12.5 ± 9.3	13.53 ± 9.6	9.5 ± 7.7	**<0.001**
Generalized Anxiety Disorder 7-item (GAD-7) (%)				
No anxiety	125 (29.2)	82 (26.0)	43 (38.1)	0.115
Mild anxiety	146 (34.1)	111 (35.2)	35 (31.0)	
Moderate anxiety	102 (23.8)	79 (25.1)	23 (20.4)	
Severe anxiety	55 (12.9)	43 (13.7)	12 (10.6)	
GAD-7 total score	8.0 ± 5.1	8.4 ± 5.0	7.1 ± 5.1	**0.011**

The data are presented as the means (±standard deviations) or counts (percentages). BDI-II: Beck Depression Inventory, second version; BMI: body mass index; GAD: generalized anxiety disorder; MEDAS: Mediterranean diet adherence screening tool; MedDiet: Mediterranean diet. ^a^ Sum of the z score of handgrip weight/weight and the z score of the standing long jump test. *p* values marked with bold indicate statistically significant differences (*p* < 0.05) between the categories of adherence to the MedDiet.

**Table 2:** Partial correlations between the MEDAS score and fat mass, lean mass, muscular strength (MSI, handgrip strength, and standing long jump), and depression (BDI-II) and anxiety (GAD-7) scores.

	Fat Mass	Lean Mass	MSI ^a^	Handgrip Strength	Standing Long Jump	Depression	Anxiety
MEDAS score	0.016	0.113 *	0.118 *	0.097 *	0.119 *	−0.223 **	−0.118 *
Fat mass	-	0.265 **	−0.199 **	−0.033	−0.334 **	0.205 **	0.046
Lean mass		-	0.737 **	0.824 **	0.527 **	−0.108 *	−0.209 **
MSI ^a^			-	0.919 **	0.917 **	−0.246 **	−0.253 **
Handgrip strength				-	0.686 **	−0.180 **	−0.236 **
Standing long jump					-	−0.272 **	−0.228 **
Depression						-	0.695 **

Adjusted by gender, age, total energy, alcohol consumption, tobacco consumption, and level of parental education. The values are the correlation coefficients. * *p* < 0.05; ** *p* < 0.001. BDI-II: Beck Depression Inventory, second version; GAD: generalized anxiety disorder; MEDAS: Mediterranean diet adherence screening tool; MSI: muscle strength index. ^a^ Sum of the standardized *z* scores of dynamometry and standing long jump.

## Data Availability

Data will be available upon reasonable request to the corresponding author. Data are not publicly available due to privacy.

## Supplementary Materials

The following supporting information can be downloaded at: https://www.mdpi.com/article/10.3390/nu17020346/s1, Table S1: Descriptive characteristics by field of studies.
