# Supplementary material for: The Association Between Adherence to the Mediterranean Diet and Depression and Anxiety Symptoms in University Students: The Mediating Role of Lean Mass and the Muscle Strength Index
Source: Nutrients. 2025 Jan 18;17(2):346. doi: 10.3390/nu17020346 (PMC11768187; doi:10.3390/nu17020346)
Supplement: Supplementary file 1 [file nutrients-17-00346-s001.zip › nutrients-3424208-supplementary.pdf]

Table S1. Descriptive characteristics by field of studies

	**Health sciences (nursing) (n = 196)**	**Non-health sciences (other university degrees) (n = 232)**
Age (years)	21.3 ± 3.1	20.7 ± 2.9
BMI (kg/m^2)	22.5 ± 3.7	23.7 ± 4.8
Muscle Strength Index (MSI)[a]	0.16 ± 1.7	-0.05 ± 1.7
Handgrip strength (kg)	26.3 ± 8.5	26.1 ± 8.6
Standing long jump (cm)	137.1 ± 33.4	137.1 ± 34.2
MEDAS score (0-14)	7.5 ± 1.8	7.2 ± 1.8
BDI-II Total score	**12.0 ± 8.4**	**13.7 ± 9.8**
GAD-7 Total score	7.8 ± 5.2	8.2 ± 4.9

The data are presented as the means (± standard deviations) or counts (percentages). BDI-II: Beck Depression Inventory, second version; BMI: body mass index; GAD: generalized anxiety disorder; MEDAS: Mediterranean diet adherence screening tool. [a] Sum of the z score of handgrip weight/weight and the z score of the standing long jump test. *P* values marked with bold indicate statistically significant differences ($p < 0.05$) between the categories
